# Methods for assessing the quality of mammalian embryos: How far we are from the gold standard?

**DOI:** 10.5935/1518-0557.20160033

**Published:** 2016

**Authors:** José C. Rocha, Felipe Passalia, Felipe D. Matos, Marc P. Maserati Jr, Mayra F. Alves, Tamie G. de Almeida, Bruna L. Cardoso, Andrea C. Basso, Marcelo F. G. Nogueira

**Affiliations:** 1 Department of Biological Science, Faculty of Sciences and Languages, São Paulo State University (UNESP); 2 Institut de Biologie de l École Normale Supérieure de Paris, Paris, France; 3 In Vitro Brasil SA - Mogi Mirim, Brazil

**Keywords:** Embryo quality assessment, morphological evaluation, artificial intelligence, mammalian embryo

## Abstract

Morphological embryo classification is of great importance for many laboratory techniques, from basic research to the ones applied to assisted reproductive technology. However, the standard classification method for both human and cattle embryos, is based on quality parameters that reflect the overall morphological quality of the embryo in cattle, or the quality of the individual embryonic structures, more relevant in human embryo classification. This assessment method is biased by the subjectivity of the evaluator and even though several guidelines exist to standardize the classification, it is not a method capable of giving reliable and trustworthy results. Latest approaches for the improvement of quality assessment include the use of data from cellular metabolism, a new morphological grading system, development kinetics and cleavage symmetry, embryo cell biopsy followed by pre-implantation genetic diagnosis, zona pellucida birefringence, ion release by the embryo cells and so forth. Nowadays there exists a great need for evaluation methods that are practical and non-invasive while being accurate and objective. A method along these lines would be of great importance to embryo evaluation by embryologists, clinicians and other professionals who work with assisted reproductive technology. Several techniques shows promising results in this sense, one being the use of digital images of the embryo as basis for features extraction and classification by means of artificial intelligence techniques (as genetic algorithms and artificial neural networks). This process has the potential to become an accurate and objective standard for embryo quality assessment.

## INTRODUCTION

Since the development of the first successful techniques for assisted reproduction in mammals, it has become evident that there is a direct relationship between embryo quality and gestational success post embryo transfer (Lindner & Wright, 1983; Overström, 1996). Embryos morphologically classified as higher quality had higher successful gestation rates in domestic animals (Schneider *et al*., 1980; Tervit *et al*., 1980; Lindner & Wright, 1983) and in human patients (Balaban *et al*., 2000, 2006; Gardner & Schoolcraft., 1999). Although the direct relationship between embryo quality and success rate based on embryo grading is clear, it is still largely subjective due to low repetitiveness, with a high grading variance between embryologists (Lindner & Wright, 1983; Farin *et al*., 1995; Richardson *et al*., 2015). Thus, there is still great need for a system capable of categorizing embryos according to quality and according to viability and the capacity for successful gestations.

Currently, for morphological classification of cattle embryos, the usual approach is the grading within three quality ranks: Excellent or good (1), regular (2) or poor (3)(Bó & Mapletoft, 2013). This method is recommended by the International Embryo Transfer Society (IETS) over the deprecated four grading systems (Lindner & Wright, 1983), which separates excellent and good embryos, and it was common before studies had shown that there is not a significant difference in gestation rates between excellent and good embryos. However, it is noteworthy that the human eye is capable of distinguishing at least four morphological quality categories of embryos. Although the current grading system is simplified to only 3 possible ranks, the embryologist should be prepared to distinguish between excellent and good quality embryos, at one time both considered part of grade 1 in the IETS system.

In the case of human embryos, the prevailing system is the one proposed by Gardner & Schoolcraft (1999), although alternative grading systems exist (Dokras *et al*., 1993; Richardson *et al*., 2015). Altogether, the simpler grading systems (Dokras *et al.*, 1993; Richardson *et al*., 2015) are more uniform and have a smaller variance between examiners. According to Balaban *et al.* (2006), the Gardner & Schoolcraft system, although more complex and with lower repetitiveness, results in higher predictive value for clinic pregnancy when compared to the proposal of Dokras *et al.* (1993). From this analysis derives an outcome that the more complex the system is, the more likely it is to grasp the biological reality of the grading system, here termed "embryo quality". Although a more straightforward system may have lower prediction accuracy, by reducing the amount of variables the system is less prone to differences between examiners, thus being more consistent.

A common factor between the systems described above is that all of them are based on the visual analysis of the embryo which is both subjective and qualitative and commonly done by stereomicroscopy. The technical quality assessment relies on the experience, attention to detail and systematic approach of the examiner on analyzing the embryo, from the more evident features as dead and extruded cells, or reduction of the percentage of viable cells to the more subtle characteristics that may influence embryo development such as irregularity of shape, heterogeneity of color, asynchrony between expected and encountered stage of development and the presence of vacuoles. On this classical approach of embryonic morphology, the variables are not measured in an objective form, resulting in low repeatability and subjectiveness of analysis (Bényei et al., 2006; van Loendersloot *et al*., 2014; Perkel *et al*., 2015; Richardson *et al*., 2015; Thompson *et al*., 2016). On this approach, a given embryo when analyzed by different examiners may be classified in different distinct degrees of quality (Farin *et al*., 1995, 1999; Chen *et al*., 2016). This variation between examiners is even more expressive between close quality grades as excellent/good and regular or regular and poor when compared to grades that are more distant, as excellent/good and poor (Farin *et al*., 1995). Additionally, the highest level of agreement between examiners occurs on the extreme classes (excellent or poor), being that the intermediate embryos are mostly responsible for the disagreement between examiners. Studies had also analyzed the effects of consecutive evaluations by the same person, so as to enable measuring the consistency (repeatability) of the evaluation (Arce *et al*., 2006; Paternot *et al*., 2009). Richardson *et al.* (2015) reported, for the classification of human embryos, a higher discrepancy between different examiners (K=0.63; Fleiss-Kappa statistic) than with the same examiner (K=0.71).

Seeking solution for the subjectivity of the morphological analysis, several alternative methods have been proposed (Overström, 1996; Hoshi, 2003; López-Damián *et al*., 2008; Held *et al*., 2012). Among them, the quality of in vitro growth of embryos, the integrity of blastomere membrane (Overström, 1996), analysis of embryo metabolism (Rondeau *et al*., 1995; Overström, 1996; Thompson *et al.*, 2016), measurement of cellular respiration (Hoshi, 2003), electron-microscopy analysis (López-Damiánet *et al*., 2008) and zona pellucida birefringence index (Heldet *et al.*, 2012). More recently, and specially for human embryos, there was a trend for methods that evaluate embryo kinetics and cleavage symmetry using time-lapse systems like EmbryoScope^®^ or Primo Vision^TM^ (Montag *et al*., 2013; Kovacs, 2014; VerMilyea *et al*., 2014). This kind of system allows the measurement of an index that stands as a guideline to aid embryologists on the selection of the best embryo for transfer in fertilization clinics. Nevertheless, for the in vitro production of cattle embryos, such a system is not widely used, mainly because of logistic limitations, the high operational cost and the reduced significance of evaluation for individual embryos. A distinct approach on early development is the classification by means of dedicated semi-automatic software (Santos Filho *et al*., 2012; Matos *et al*., 2014a), in a way that the analysis is not dependent on specific hardware. Table 1 shows a broader comparison among the different methods proposed for the morphological classification of human, cattle and murine embryos.

**Table 1:** Studies addressing methods to evaluate mammalian embryos by non-invasive or invasive techniques.

Authors	Technique	Nature	Species	Embryo stage	Compared to what traditional technique	On what parameters	Pros	Contras	Sample Size
Lindner & Wright, 1983	Morphological assessment by stereomicroscopy	Noninvasive	Bovine	From morula to hatched blastocyst	Physiological parameters of devel-opment kinetics and pregnancy rates	Embryo stage and quality (excellent, good, fair and poor) on pregnan-cy rates	Embryo quality was a useful predictor of the pregnancy rate	Subjectivity of the embryo quality assessment; excellent and good qualities had no difference on pregnancy rate	783
Gardner & Schoolcraft, 1999	Morphological assessment by stereomicroscopy	Noninvasive	Human	Blastocyst	Morphological aspects of the trophectoderm and inner cell mass	Blastocyst quality based on grades	Validated worldwide by embryologists and clinicians due its useful relation with pregnancy establishment	Does not cover all aspects of the aberrant morphology and it is limited to blastocyst stage	ND
Balaban *et al*., 2006	Morphological assessment by stereomicroscopy	Noninvasive	Human	Blastocyst	Comparison between the Gardner and Dokras systems of evaluation	Clinical pregnancy, implantation rate and multiple pregnancies	Both systems were practical and accurate, good to predict blastocysts with high implantation potential and to limit the number of embryo transfer to avoid multiple pregnancies	Lack of a single and unified system and a standard to evaluate	132
Richardson *et al*., 2015	Simplified method to evaluate blastocysts	Noninvasive	Human	Day 5 blastocyst	Traditional method to evaluate embryo morphology	New and simpli-fied method with less parameters to assess	Faster and easier than the traditional method (Gardner & Schoolcraft, 1999) with similar or better results than a more complex system	Method rarely used by clinicians	80
Shiku *et al*., 2001	Scanning electrochemical microscopy (SECM)	Noninvasive	Bovine	From morula to blastocyst	NA	Results showed that morulae with higher oxygen consumption were faster to develop into blastocysts. The method could be just a complement or to change the way how the embryo is chosen	Noninvasive technique that could be reliable to the embryo viability assessment and development	Not well established and worldwide acceptation	19
Braga *et al*., 2015	Mass spectrometry to charac-terize (“fin-gerprint”) the culture medium conditioned by the embryo	Noninvasive	Human	Day 3 of culture	Spectroscopy	To identify potential embryo lipid biomarkers that are predictors to preview blastocyst formation	Promising approach to identify embryos that should be cultured until Day 5 or cryopreserved		50
Yang *et al*., 2012	Chromosomal screening by array Comparative Genomic Hybridization (aCGH)	Invasive	Human	Day 5	NA	Together with morphological evaluation presented the best results	Produced clinical pregnancy more frequently and a lower abortion rate when compared to embryos chosen without a CGH	Demands skills from the embryologist and complex equipment to perform the invasive technique	814
Kropp *et al*., 2014	Micro RNA (miRNA) profile	Noninvasive	Bovine and Human	Day 5 or 6	NA	Potential to develop noninvasive biomarkers to predict embryo quality	Embryo quality related with some miRNA expression	Needs more studies and development of robust and accurate biomarkers	216 (bovine embryos)
Kakourou *et al*., 2013	Transcriptomic analysis	Invasive	Human	Blastocyst	NA	Results highlighted the importance of the hormones and their receptors but lack a physiologic comprehension of their role on the early development	Future analyses could identify new biomarkers that predict embryo development potential	Still an experimental method that needs more studies	03
Farin *et al*., 1995	Videotape assessment of embryo images	Noninvasive	Bovine	From morula to blastocyst	NA	Way to measure interembryologist agreement. Quality	Good to excellent agreement existed for classifying Day 7 embryos by stage and by extremes of quality grade (grades 1 and 4). It was proposed a simple grading criteia to maximize agreement among evaluators	There was poor agreement of evaluators by degree of abnormal morphology (Grades 2 and 3)	40
VerMilyea *et al*., 2014	Timelapse	Noninvasive	Human	Day 1 to 3	NA	Strong predic-tion of the clinical pregnancy when related to P2 (time between 1st and 2nd mitosis or 2 cellstage duration) and P3 (time between 2nd and 3rd mito-sis or 3 cell-stage duration)	Automatized predictive model	Not suitable to blastocyst evaluation	375
Milewski *et al*., 2015	Timelapse	Noninvasive	Human	Day 5	NA	Based on time of cell divisions (2 and 5 blastomeres) and inter-val between 2nd and 3rd divisions, it was proposed a multivariate predictive model	Evaluation of morphokinetics parameters could provide data encompassing a long time frame of embryo development. This is the main advantage of the method, i.e. the opportunity to observe the embryo almost continuously	The method considers only morphokinetic parameters - such as the relative and absolute times of cell division – on the impact of the embryo capacity to reach the blastocyst stage	162
Wong *et al*., 2010	Image analysis algorithm	Noninvasive	Human	Day 2	NA	First algorithm attempt to ebryo development automatized analysis. Could predict the blastocyst stage of develop-ment	Automatized analysis of parameters	Unable to discriminate parameters to predict the development up to blastocyst and its quality	242
Santos Filho *et al*., 2012	Support Vector Machine	Noninvasive	Human	Blastocyst	NA	Potential method to automatized embryo classification discriminating quality parameters of inner cell mass and trophectoderm	Method based only on the blastocyst image (numerical data mined from it with more discrimina-tory parameters than visual morphology assessment by human beings)	Semiautomatic meth-od was obtained and a fully automatized meth-od should be achieved	73
van Loender-sloot *et al*., 2014	Logistic regression	Noninvasive	Human	Day 3	Embryonic morphology	Early cleavage, blastomere number on days 2 and 3, morphologic pointing and presence of a morula no Day 3 of culture	Method capable to distinguish embryos with high implantation potential from those with moderated or low potential		6021
Chen *et al*., 2016	Data mining to produce a computer assisted scoring system based on multivariable logistic regression and multivariate adaptative re-gression spline	Noninvasive	Human	Day 1,2 and 3	Embryonic morphology	Point grading system	Improvement on the generalization of the current predictive models	Promising approach although still experimental	871
Matos *et al*., 2014a	Artificial neural network	Noninvasive	Mouse	Blastocyst	Embryonic morphology	To avoid the subjectivity of the assessment done by a human examiner	Classification systems fully based on software	Needs an embryologist to manually to obtain numerical parameters from the embryo image	98
Matsuura *et al*., 2010	Blastocyst quality score	Noninvasive	Human	Blastocyst	NA	New pointing system	Numerical classifi-cation system based on the Gardner & Schoolcraft (1999) criteria	There is a requirement for a huge number of embryos to produce statistical significance	220
Tejera *et al*., 2012	Oxygen consumption measurement	Noninvasive	Human	Day 3	NA	Oxygen consump-tion rate was associated with potential implantation and embryo quality	Technique used as a complementary pa-rameter to determine the embryo to be chosen	Not useful to predict implantation rates. The causes of this lack of predictability is the clinical relevance of other variables that are related to embryo quality	84
Thompson* et al.*, 2016	Multi-spectral imaging to evaluate the endogenous auto fluores-cence	Noninvasive	Human and Bovine	Early embryo stages	Diversity of techniques	The authors are trying to correlate the observed pattern of auto fluorescence with metabolic profile of the embryo. The aim is to predict the embryo quality during development of early embryos	High resolution imag-ing (single embryo), real time and noninvasive method that could be associated with others (e.g., tra-ditional morphological evaluation) besides computer based techniques. It is a current promise to determine the intracellular met-abolic activity	Experimental technique under evaluation	ND
López-Damián *et al*., 2008	Transmission electron microscopy	Lethal	Bovine	Blastocyst	NA	Demonstrated the sub-cellular varia-tion of the embryos classified as fair grade by optical light microscopy and by stereomi-croscopy	Validated the ac-curacy of the IETS proposed system of embryo classification	Unable to evaluate an embryo intended to be transferred to the uterus, just to validate the accuracy of a technique on subcellular aspects	30

Legend: Nature - invasiveness of the technique employed; Embryo stage – range of stages where the embryo was suitable or was used in the study; Compared to what traditional technique – when it was the case - that a new proposed technique had its efficacy compared to a traditional and well established one; On what parameters – when there was such comparison, did it occur, if not the case, in what primary parameters the authors evaluated the embryos; Sample size – the quantity of embryos used on the study; NA – not available; ND – not determined.

Still, no method thus far has been able to reach a definitive solution for the measurement of embryo quality, considering that many are still in experimental stage. Therefore, the research and development of techniques that prove to be fast, non-invasive and objective are fundamental in the development of any embryo grading system (Lindner & Gardner, 1983; Overström, 1996; Thompson *et al*., 2016). While for some methods the limiting factor is the high cost of implementation, preventing use on different species of mammals (time-lapse analysis, biopsy followed by pre-implantation genetic diagnosis) for others, the invasiveness - or even the lethality, as with the ultra-structural analysis (López-Damiánet *et al.*, 2008) - is the crucial point. Thus, regardless of subjectivity, visual analysis of embryo morphology is still generally used to determine embryo quality.

Several authors recently proposed the use of mathematical and statistical tools for the analysis of embryo viability. Among the main researches, van Loendersloot *et al*. (2014) reported the use of multivariate logistic regression with eight predictive factors for the classification of embryos according to implantation potential. Such a model has shown a moderate discriminative capacity, being able to categorize embryos with high, moderate or low implantation potential. Nevertheless, we need to stress that the method also uses other variables rather than embryo morphology, such as physiological, endocrinological and metabolic parameters of the patient who will receive the embryo.

Chen *et al.* (2016) proposed the use of a computer-assisted scoring system (CASS). The system is supposed to have a higher discriminatory power for embryo selection, over the standard scoring system that has intrinsic examiner variability. The authors also used a multivariate logistic regression (LR) system, together with multivariate adaptive regression splines (MARS). The study had shown improvement on the predictive model when using the computer assisted scoring system associated with data mining.

Santos Filho *et al*. (2012) developed a system, by means of applied mathematics, capable of acting in a semi-automatic fashion on the interpretation and classification of human embryos. Such a proposal proved to be unique and managed to overcame an innovative challenge as no similar technique with comparable results exists. In this way, the fact that the process is not fully automated and only aimed at human embryo evaluation limits the diffusion of the methodology to other species and to practical routine laboratory work.

More recently, another group published research on embryo viability grading using image processing techniques based on the segmentation of blastomeres (Singh *et al*., 2014; Tian *et al*., 2014) or trophectoderm (Singh *et al*., 2015) from human embryos.

### Artificial intelligence as a new way to approach the problem

Artificial intelligence (AI) techniques have the potential to develop objective, reproducible and non-invasive methodologies to predict embryo quality with high accuracy. The field of AI is very extensive, but some specific techniques as genetic algorithms (GA) along with artificial neural network (ANN) could be used to simulate an accurate predictive model (Takahashi *et al.*, 2016).

GA is a search and optimization method inspired by genetic mechanisms and natural evolution. In GA a population of possible solutions is simulated for a determined problem, that is, a population of 'individuals' each one containing a possible solution. By an evolutionary process based on crossover, mutations and migrations, the individuals can converge to a better solution for the problem (Tanomaru, 1995). ANN is a technique based on how human neurons transmit and process information and it is indicated for the resolution of complex and nonlinear problems. Such neurons need to be exposed to training data (variables), in order to learn to generalize an output (i.e., a result) from a input dataset. Once properly trained, ANN is able to perform predictions from new input data to which it has never has access (Haykin, 1998; Zhang *et al*., 1998; Huang, 2009).

Initially proposed for mouse embryos (Matos *et al*., 2014a) and posteriorly applied in bovine blastocysts (Matos *et al.*, 2014b), this potential method uses a process of automated extraction of information, from bi-dimensional digital images of embryos and, posteriorly, classifies them in quality grades, according to the specificity of each species. These two cases in particular used just blastocysts between the initial and expanded stages. The blastocyst stage is the standard in commercial procedures of bovine embryos transfers, produced in vitro, as well as has been increasingly used for clinical procedures in assisted human reproduction laboratories (Balaban *et al*., 2000; Hyttel *et al*., 2010).

**Figure 1:**
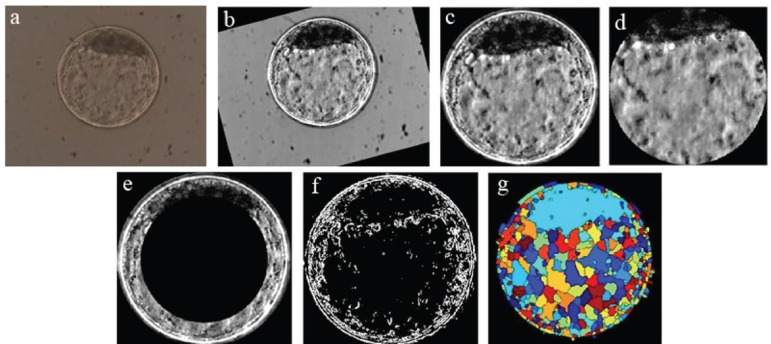
Ilustrates the sequence of steps required to process a digital image from an in vitro produced bovine blastocyst. a) original image as obtained by optic microscopy; b) standardization of bright and positioning of the inner cell mass (ICM) at 12 o’clock; c) segmentation of the embryo itself (by Hough transform) and elimination of background; d) segmentation of ICM and blastocoel by elimination of the zona pellucida and trophectoderm; e) elimination of inner area of the image “c” to highlight the trophectoderm and part of the ICM; f) binary form of image “c” after gradient calculation; g) visualization of the image after Watershed transform.

In a paper published by Matos *et al.* (2014b), blastocyst digital images were captured by optic microscopy without the use of dye while maintaining embryo exposure lower than 30 seconds, using techniques of digital image processing (Gonzalez & Woods, 2008) for information standardization and interpretation. Once the embryo was properly standardized and isolated of its background (in an automated mode), it was possible to do a segmentation step, that is, the extraction of several numeric variables contained in the digital image. Thus, these variables obtained were used as input to the ANN system. The objective of the information extraction is to obtain a numeric vector, which represents the original image. Several algorithms work independently in this process, providing the input variables to the ANN. Therefore, we used techniques such as Hough transform (Atherton & Kerbyson, 1999) to determine embryo circularity, texture analysis (Haralick *et al*., 1973; Tuceryan & Jain, 1998; Soille, 2013; Sonka *et al*., 2014) using the Gray Level Co-occurrence Matrix(GLCM) classification method (Hu *et al.*, 2008; Siqueira *et al*., 2013) and the Watershed transform (Beucher, 1992), that proposes a morphologic approach to the problem of image segmentation, by its interpretation as being surfaces, in which the grey levels of each pixel determines the altitude of a given region (Körbes, 2010). Figure 1 ilustrates the sequence of steps used to process a digital image from an in vitro produced bovine blastocyst.

All the possible information from the digital images of bovine blastocysts was extracted, and 36 variables were obtained to define the embryo (i.e., the mathematic representation of the main features of the digital image). After a co-linearity analysis, these 36 variables were reduced to 24, which were used as the input data for ANN. After training, these variables made up the GA population. This has undergone the natural evolution process (containing the crossover, mutation and migration events) which determined the most suitable ANN for the embryos classification.

In results obtained recently (not published) of our research group, and involving 126 images of bovine blastocysts, after three experienced embryologists analysed the images, the results were applied to the GA technique associated to ANN. As the network output standard (template) was used, the mode value of the classification was made by the embryologists. Seventy percent of the sample was utilized for training and 15% for ANN validation leaving 15% for testing the system. The result in a blind test with the 15% remaining resulted in 84% correct in exact classification of embryos, that is, the ANN classified with the same mode value given by the trained embryologists. In this blind test there were no detected critic error in evaluation by ANN, that is, the cases in which ANN classified the image in a grade than the one rated by the examiners (e.g., the examiners classified the image as excellent and the ANN as poor). Therefore, we consider the accuracy of the applied method for embryo classification as satisfactory, showing to be a promising technique with potential for clinical application.

Our study, which is still in the experimental stage and in collaboration with the world's largest company of bovine embryo in vitro production (In Vitro Brasil, Mogi Mirim, SP, Brazil), is protected by a national patent application filling with INPI (BR102012031953-5; Matos *et al*., 2016) and international with WIPO (PCT-BR2013-000506; Matos *et al.*, 2014c), in which both were done together with Agência Unesp de Inovação (AUIN). This is, to our knowledge, the only other registered invention engaged in embryo selection (Loewke & Suraj, 2014). However, our invention differs from Loewke & Suraj's (2014), by the use of a time-lapse image acquisition system for determining embryo quality, which is based on the kinetics and symmetry of embryo cleavage. We infer that both classification systems are not mutually exclusive. The kinetic evaluation and symmetry as well as the blastocyst image by ANN could be made available in hardware by time-lapse video equipment.

## CONCLUSION

In light of the multiple current attempts to develop a precise non-invasive system for embryo classification, this is still an ongoing process. Clinicians and researchers are waiting for a system that is non-invasive, objective and accurate, for prediction and with high reproducibility. The most promising alternatives seems to be the ones that take into account the metabolites used by the embryo and obtained by analysis of the conditioned culture medium, the use of applied mathematics and statistics with the classificatory system or dedicated software for the analysis of kinetics, symmetry or morphology of the embryo. In the absence of a robust and well-established system, the majority of embryologists will continue to rely on the conventional classification system that, despite its inaccuracies, it still bears some predictive power for successful implantation and the ability to classify embryos morphologically. However, no matter how new technologies may be developed, they cannot currently surpass human evaluation with years of clinical experience on the ultimate assessment of embryo quality.

Finally, we foresee the possibility of an artificial intelligence system, similar to the one described before, but not limited only to the morphological analysis of the embryo. Theoretically, it is possible to adapt the system for the direct prediction of successful embryo implantation, once the variables that describe the physiological, endocrinological and metabolic environment of the recipient are included on the machine learning algorithms.
